# Associations of fetal and postnatal growth trajectories with child cognition: the GUSTO cohort study

**DOI:** 10.1093/ije/dyaf012

**Published:** 2025-02-13

**Authors:** Yi Ying Ong, Nicholas Beng Hui Ng, Navin Michael, Shirong Cai, Mya Thway Tint, Delicia Shu Qin Ooi, Ai Peng Tan, Kok Hian Tan, Lynette Shek, Fabian Yap, Yap Seng Chong, Johan Gunnar Eriksson, Shiao-Yng Chan, Birit F P Broekman, Keith M Godfrey, Patricia Pelufo Silveira, Henning Tiemeier, Evelyn C Law, Izzuddin M Aris, Yung Seng Lee

**Affiliations:** Department of Paediatrics, Yong Loo Lin School of Medicine, National University of Singapore, Singapore, Singapore; Department of Social and Behavioral Sciences, Harvard T.H. Chan School of Public Health, Boston, MA, United States; Department of Paediatrics, Yong Loo Lin School of Medicine, National University of Singapore, Singapore, Singapore; Department of Paediatrics, Khoo Teck Puat—National University Children’s Medical Institute, National University Health System, Singapore, Singapore; Singapore Institute for Clinical Science, Agency for Science, Technology, and Research, Singapore, Singapore; Singapore Institute for Clinical Science, Agency for Science, Technology, and Research, Singapore, Singapore; Singapore Institute for Clinical Science, Agency for Science, Technology, and Research, Singapore, Singapore; Department of Paediatrics, Yong Loo Lin School of Medicine, National University of Singapore, Singapore, Singapore; Singapore Institute for Clinical Science, Agency for Science, Technology, and Research, Singapore, Singapore; Department of Diagnostic Imaging, National University Health System, Singapore, Singapore; Office of Academic Medicine, Duke-NUS Medical School, Singapore, Singapore; Department of Maternal Fetal Medicine, KK Women’s and Children’s Hospital, Singapore, Singapore; Department of Paediatrics, Yong Loo Lin School of Medicine, National University of Singapore, Singapore, Singapore; Department of Paediatrics, Khoo Teck Puat—National University Children’s Medical Institute, National University Health System, Singapore, Singapore; Singapore Institute for Clinical Science, Agency for Science, Technology, and Research, Singapore, Singapore; Division of Paediatric Medicine, KK Women’s and Children’s Hospital, Singapore, Singapore; Singapore Institute for Clinical Science, Agency for Science, Technology, and Research, Singapore, Singapore; Department of Obstetrics and Gynaecology and Human Potential Translational Research Programme, Yong Loo Lin School of Medicine, National University of Singapore, Singapore, Singapore; Singapore Institute for Clinical Science, Agency for Science, Technology, and Research, Singapore, Singapore; Department of Obstetrics and Gynaecology and Human Potential Translational Research Programme, Yong Loo Lin School of Medicine, National University of Singapore, Singapore, Singapore; Department of General Practice and Primary Health Care, University of Helsinki, Helsinki, Finland; Public Health Research Program, Folkhälsan Research Center, Helsinki, Finland; Singapore Institute for Clinical Science, Agency for Science, Technology, and Research, Singapore, Singapore; Department of Obstetrics and Gynaecology and Human Potential Translational Research Programme, Yong Loo Lin School of Medicine, National University of Singapore, Singapore, Singapore; Department of Psychiatry, OLVG and Amsterdam UMC, Amsterdam Public Health Institute, Vrije Universiteit, Amsterdam, The Netherlands; MRC Lifecourse Epidemiology Centre and NIHR Southampton Biomedical Research Centre, University of Southampton and University Hospital Southampton NHS Foundation Trust, Southampton, UK; Department of Paediatrics, Yong Loo Lin School of Medicine, National University of Singapore, Singapore, Singapore; Ludmer Centre for Neuroinformatics and Mental Health, Department of Psychiatry, McGill University, Montreal, QC, Canada; Douglas Mental Health University Research Centre, Montreal, QC, Canada; Department of Social and Behavioral Sciences, Harvard T.H. Chan School of Public Health, Boston, MA, United States; Department of Paediatrics, Yong Loo Lin School of Medicine, National University of Singapore, Singapore, Singapore; Department of Paediatrics, Khoo Teck Puat—National University Children’s Medical Institute, National University Health System, Singapore, Singapore; Singapore Institute for Clinical Science, Agency for Science, Technology, and Research, Singapore, Singapore; Division of Chronic Disease Research Across the Lifecourse, Department of Population Medicine, Harvard Medical School and Harvard Pilgrim Health Care Institute, Boston, MA, United States; Department of Paediatrics, Yong Loo Lin School of Medicine, National University of Singapore, Singapore, Singapore; Department of Paediatrics, Khoo Teck Puat—National University Children’s Medical Institute, National University Health System, Singapore, Singapore; Singapore Institute for Clinical Science, Agency for Science, Technology, and Research, Singapore, Singapore

**Keywords:** fetal growth, postnatal growth, body mass index, weight gain, growth trajectories, cognition, neurodevelopment, intelligence, early childhood, developmental origins of health and disease

## Abstract

**Background:**

Using longitudinal ultrasounds as an improved fetal growth marker, we aimed to investigate if increased postnatal growth following fetal abdominal circumference (AC) growth deceleration is associated with improved child cognition.

**Methods:**

Among 797 term-born singletons in the Growing Up in Singapore Towards healthy Outcomes (GUSTO) cohort, we derived 2nd–3rd trimester fetal AC growth z-score, fetal AC growth deceleration, standardized height, weight, and body mass index (BMI) growth at early infancy (0–4 months), late infancy (4–15 months), toddlerhood (15–37 months), and early childhood (3–7 years), and investigated their associations with intelligence quotient (IQ) at ages 4.5 years (verbal, non-verbal) and 7 years (non-verbal—block design, matrix reasoning), adjusting for socio-demographic and biological confounders.

**Results:**

Among term-born newborns, 23.3% experienced fetal AC growth deceleration, which was associated with lower non-verbal IQ (4.5 years) [β (95% CI), –4.00 (–7.49, –0.51)]. Higher 0–7 years z-BMI gain was associated with lower non-verbal IQ (block design) (7 years) [–1.33 (–2.51, –0.14)]. Higher late infancy z-BMI gain was associated with higher verbal IQ (4.5 years) [3.36 (0.82,5.90)] but lower non-verbal IQ (matrix reasoning) (7 years) [–2.32 (–4.48, –0.17)]. Among those with fetal AC growth deceleration, higher 0–7 years z-weight gain was associated with lower non-verbal IQ (block design) (7 years) (*P*-interaction = .049); at z-weight gain of +2 standard deviation score (SDS), those with fetal AC growth deceleration had lower IQ [margins (95% CI), –2.6 (–7.1,1.9)]. On average, children with fetal AC growth deceleration caught up in z-height, z-weight, and z-BMI by 7 years.

**Conclusion:**

Fetal AC growth deceleration was associated with lower cognition scores at preschool age. Increased weight or BMI growth from 0–7 years following fetal AC growth deceleration might not be favorable to cognition among generally well-nourished term-born children.

Key Messages

- While previous studies focused on low birthweight, small-for-gestational age, or preterm infants, we sought to investigate if there are unidentified groups of term-born infants at risk of poor neurodevelopment by studying their fetal and postnatal growth trajectories on cognition scores in childhood.
- Fetal abdominal circumference (AC) growth deceleration from the second to third trimester, observed in 23.3% of term-born infants, was associated with lower cognition scores at preschool age but not at school age—on average, these children also had a gradual ‘catch-up’ in postnatal growth and have caught up in growth by school age.
- Excessive postnatal weight gain in children with fetal AC growth deceleration did not benefit cognition and may be associated with lower non-verbal IQ scores, suggesting that early identification to manage excessive weight gain should be considered.

## Introduction

Poor fetal growth, which occurs when a fetus is unable to attain its growth potential due to suboptimal *in utero* environment [1, 2], has been associated with suboptimal neurodevelopmental outcomes [3, 4]. Small-for-gestational age at birth, often used as a proxy of poor fetal growth, does not differentiate constitutionally small newborns who had appropriate fetal growth from newborns who had experienced fetal growth deceleration (deviation from their growth potential) [1, 2]. To overcome this, recent studies have investigated longitudinal fetal abdominal circumference (AC) growth, which reflects nutritionally sensitive compartments such as the liver and abdominal fat, and found that it was associated with feto–placental blood flow, neonatal morbidity, child vision, and child cardiometabolic markers [5–9]. However, associations between fetal AC growth deceleration and child cognition remains poorly established. Furthermore, while postnatal catch-up growth was associated with improved neurocognitive outcomes among preterm, low birthweight, or small-for-gestational age (SGA) infants [10–12], it is unknown if these associations hold among term-born infants with fetal AC growth deceleration. Few studies investigated sensitive periods of postnatal growth [13] and their potential interactions with poor fetal growth on child cognition, which might be important for targeted monitoring.

To address these gaps, we aimed to investigate associations of longitudinal ultrasound-measured fetal AC growth, period-specific postnatal growth (early infancy, late infancy, toddlerhood, early childhood), and their potential interactions with child cognition. We hypothesized that children who experienced fetal AC growth deceleration have altered postnatal growth trajectories and lower cognition scores, especially if they did not have postnatal catch-up growth [12].

## Methods

### Study population

We studied participants in Growing Up in Singapore Towards healthy Outcomes (GUSTO), an ongoing prospective cohort study described previously [14]. Briefly, between 2009 and 2010, pregnant women were recruited during their first trimester from KK Women’s and Children’s Hospital and National University Hospital in Singapore. Eligibility criteria included Singapore citizens/permanent residents aged at least 18 years, of homogenous parental ethnic background; exclusion criteria included being on chemotherapy, psychotropic drugs, or having type 1 diabetes. Of 1450 participants recruited, we included 797 term-born singletons with at least 1 outcome measurement (Supplementary Fig. S1).

### Exposures

We measured fetal abdominal circumference (AC) at the second trimester (mean gestational age: 20.3 ± 0.8 weeks) and third trimester (mean gestational age: 32.9 ± 0.8 weeks) by ultrasound (GE Voluson 730 Expert transabdominal probe AB2–7, 2–7 MHz broadband curved array transducer or GE Voluson 730 PRO transabdominal probe 4CA, broadband curved array transducer). We derived fetal AC z-scores using INTERGROWTH-21st standards [15]. Based on prior literature, we defined fetal AC growth deceleration as a change in fetal AC z-scores from the second to third trimester by ≤ –0.67 standard deviation score (SDS), which represents a biologically significant downward crossing of at least one major percentile band on standard growth charts [5, 16]. This cut-point is commonly used in previous publications [5, 9, 16–19]. The continuous measure of fetal AC growth retains more information than the categorical definition of fetal AC growth deceleration, which contributes to more robust findings. However, as fetal AC growth deceleration may be useful to clinicians due to its clinically significant cut-off and ease of visualization of interactions, we present both continuous and categorical measures.

We obtained birthweight and sex of the child from medical records and determined gestational age (GA) from the first trimester dating ultrasound scan. We calculated birthweight z-scores, adjusted for sex and GA, based on customized birth charts [20] and categorized newborns as small-for-gestational-age (SGA), appropriate-for-gestational age (AGA), or large-for-gestational age (LGA).

We obtained weight and length at birth from medical records and measured child weight and length/height at postnatal visits (1 day, 1 week, 3 weeks; 3-monthly from 3–18 months; 2 years, 3 years, half-yearly from 4–7 years), using calibrated weighing scales (SECA 334; SECA 803), mobile measuring mat (SECA 210), and a stadiometer (SECA 213) (beyond 24 months). We estimated individual-level length/height, weight, and body mass index (BMI) trajectories from ages 0–7 years using linear spline mixed-effects models and selected knot positions based on Bayesian information criterion (Supplementary Table S1), described previously [21]. To enable better comparison with other studies, we modelled trajectories of length/height, weight, and BMI z-scores derived from World Health Organization growth references [22] by performing linear spline mixed-effects modelling. We derived standardized length/height, weight, and BMI growth by calculating the change in z-length/height, z-weight, and z-BMI in early infancy (0–4 months), late infancy (4–15 months), toddlerhood (15–37 months), and early childhood (37–84 months), respectively.

### Outcomes

We assessed child intelligence at the 4.5-year-old postnatal visit (mean age: 4.6 ± 0.1 years) through the Kaufman Brief Intelligence Test, Second Edition (KBIT-2), which yields verbal and non-verbal intelligence quotient (IQ) scores [23]. The verbal component consisted of the Verbal Knowledge and Riddles subtests which measured receptive vocabulary and verbal reasoning. The non-verbal component consisted of the Matrices subtest which measured fluid intelligence. At the 7-year-old postnatal visit (mean age: 7.4 ± 0.1 years), we assessed non-verbal intelligence through the Wechsler Abbreviated Scale of Intelligence, Second Edition (WASI-II)^24^—the Matrix Reasoning subtest measures fluid and visual intelligence, spatial ability, and perceptual organization; the Block Design subtest evaluates analysis and synthesis of visual stimuli, visual perception, and visual-motor coordination. We included the following secondary outcome measures at the 4-year-old visit (mean age: 4.0 ± 0.0 years) – Peabody Picture Vocabulary Test, Fourth Edition (PPVT-4) [25] which measures vocabulary knowledge; Lollipop test, a well-validated multidimensional measure of school readiness and general crystallized knowledge [26]. We derived cohort-specific standard scores for the Lollipop test with a mean of 100 and standard deviation (SD) of 15.

### Covariates

We collected socio-demographic data (mother’s age, ethnicity, highest educational attainment, total household income, parity) and self-reported pre-pregnancy weight at recruitment or the first clinic visit using structured questionnaires. At 26–28 weeks of gestation, we measured maternal height, administered questionnaires on the mothers’ smoking status and environmental (home or work) tobacco exposure, and assessed maternal depressive symptoms with the 10-item Edinburgh Postnatal Depression Scale. During the postnatal year 2 or 3 visit, we measured father’s height and weight and collected data on father’s highest educational attainment.

### Statistical analysis

We performed two-tailed *t*-tests (continuous variables) and chi-square tests (categorical variables) to compare baseline characteristics between included and excluded participants. We performed multiple linear regression to investigate associations of fetal and postnatal growth with child cognition, ensuring the linearity assumption was satisfied. We tested for non-linearity by including spline terms. In all models, we adjusted for socio-demographic (mother’s age, ethnicity, parents’ education, household income, parity, and child’s sex) and biological (parents’ height, mothers’ pre-pregnancy BMI, gestational tobacco exposure, depressive symptoms, and gestational age) confounders determined *a priori*, child’s age at cognitive assessment, whether KBIT-2 was administered in the child’s dominant language, and growth at all preceding age periods. We investigated interactions between fetal AC growth deceleration and period-specific postnatal growth by including multiplicative interaction terms. We visualized postnatal growth trajectories by including interactions of fetal AC growth deceleration with spline terms as fixed parameters in linear spline mixed effect models.

In all analyses, we used chained equation multiple imputation to impute missing covariates and exposures (*n* = 50 imputations) for 1087 eligible GUSTO participants with singleton term births. We included all exposures, covariates, outcomes, and fetal AC z-scores at gestational week 26 (auxiliary variable to improve imputation of fetal AC growth) in the imputation model. We restricted our analyses to participants with observed outcome data based on von Hippel's ‘multiple imputation, then deletion’ approach [27]. To adjust for selection bias from missing outcomes, we calculated stabilized inverse probability of censoring weights in each imputed dataset, with the denominator being the probability of being uncensored conditional on all confounders and exposures and the numerator being the probability of being uncensored conditional on exposures. We truncated stabilized weights at the 1st and 99th percentiles [28]. This approach generates a pseudo-population that reflects what would have been observed if the losses to follow-up had occurred randomly, without being influenced by the measured factors and baseline characteristics associated with loss to follow-up [29]. We combined imputed datasets using ‘mi estimate’ in Stata and fitted regression models weighted on the stabilized weights for each individual. To assess the robustness of our findings, we carried out complete case analysis, explored stricter cut-off points for defining fetal AC growth deceleration (–1 SD and –1.5 SD versus –0.67 SD), adjusted for additional potential confounders (father’s BMI), and controlled for multiple comparisons using the Benjamini–Hochberg false discovery rate (FDR) at 5% for each growth measure of interest (fetal AC growth, birthweight-for-gestational age, postnatal height, weight, BMI). We performed analyses using Stata17.0 (StataCorp LP, TX) and R (version 4.4.1). To interpret findings, we focused on the direction, magnitude, and precision of effect estimates.

## Results

### Cohort description

We included 797 children with at least 1 cognition outcome measured. Among children with IQ measured at age 4.5 years, 76% also had cognition assessed at age 7 years (Supplementary Fig. S1). Mothers had a mean age of 30.9 ± 5.0 years, 10.4% of the children were born SGA, while 23.3% experienced fetal AC growth deceleration (Table 1). Compared to included participants, excluded participants did not differ in baseline characteristics other than having lower maternal age (30.1 ± 5.2 years) (Supplementary Table S2). Compared to participants with all 3 main outcomes (IQ scores at years 4.5 and 7) measured, those without all three outcomes measured had higher father’s education, lower maternal tobacco exposure, were less likely to have depressive symptoms during pregnancy and more likely to be born AGA (Supplementary Table S3).

**Table 1.** Characteristics of participants

Characteristics	Mean ± SD/*n* (%)
Parent characteristics	
Mother’s age, years (*n* = 797)	30.9 ± 5.0
Mother’s ethnicity	
Chinese	456 (57.2%)
Malay	201 (25.2%)
Indian	140 (17.6%)
Mother’s education	
Not college graduate	504 (63.2%)
College graduate	286 (35.9%)
Data missing	7 (0.9%)
Father’s education	
Not college graduate	380 (47.7%)
College graduate	261 (32.7%)
Data missing	156 (19.6%)
Monthly household income	
Low (S$0–3999)	334 (41.9%)
Mid (S$4000–6000)	177 (22.2%)
High (>S$6000)	236 (29.6%)
Data missing	50 (6.3%)
Parity	
Primiparous	362 (45.4%)
Multiparous	435 (54.6%)
Mother’s height, cm (*n* = 779)	158.2 ± 5.7
Father’s height, cm (*n* = 734)	170.8 ± 6.2
Pre-pregnancy BMI, kg/m^2^ (*n* = 727)	22.8 ± 4.4
Maternal tobacco exposure	
No exposure	476 (59.7%)
Secondhand exposure	258 (32.4%)
Current smoker	20 (2.5%)
Data missing	43 (5.4%)
Depressive symptoms during pregnancy	
EPDS score <13	679 (85.2%)
EPDS score ≥13	92 (11.5%)
Data missing	26 (3.3%)
Child characteristics	
Sex	
Male	387 (48.6%)
Female	410 (51.4%)
Gestational age, weeks (*n* = 797)	39.0 ± 1.0
BW-for-GA z-score (*n* = 797)	0.20 ± 1.18
Appropriate-for-gestational age	585 (73.4%)
Small-for-gestational age	83 (10.4%)
Large-for-gestational age	129 (16.2%)
Fetal AC z-score change from 2nd to 3rd trimester (*n* = 743)	0.01 ± 0.99
No fetal AC growth deceleration	557 (69.9%)
Fetal AC growth deceleration	186 (23.3%)
Data missing	54 (6.8%)
IQ measures	
Verbal IQ Y4.5 (*n* = 444)	86 ± 16
Non-verbal IQ Y4.5 (*n* = 447)	100 ± 15
Non-verbal IQ Y7 (block design) (*n* = 496)	104 ± 14
Non-verbal IQ Y7 (matrix reasoning) (*n* = 496)	105 ± 14
Additional cognition measures at Y4	
Peabody Picture Vocabulary Test score (*n* = 733)	88 ± 18
Lollipop test score (*n* = 725)	100 ± 15

AC, abdominal circumference; BMI, body mass index; BW-for-GA, birthweight-for-gestational age; EPDS, Edinburgh Postnatal Depression Scale; IQ, intelligence quotient; SD, standard deviation; Y, year.

### Fetal growth

**Figure 1.**
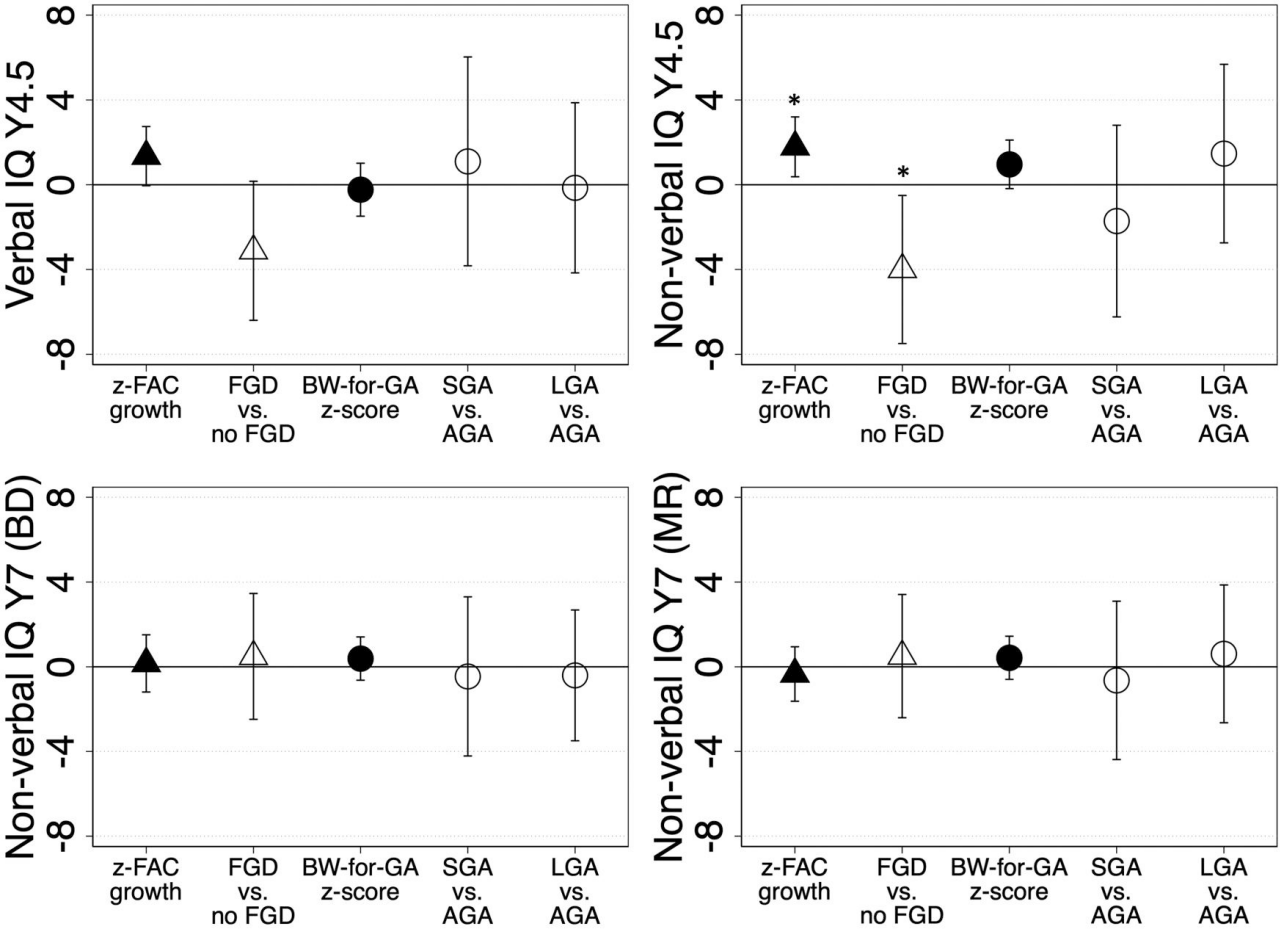
Associations between fetal growth and cognition outcomes. Models are adjusted for parents’ education, parents’ height, household income, mother’s age, parity, ethnicity, pre-pregnancy body mass index, gestational tobacco exposure, depressive symptoms, child’s sex, gestational age, and age at cognition measure. In these coefficient plots, markers represent regression coefficients, and error bars represent 95% confidence intervals, and asterisks represent *P* < .05. None of the associations had a false discovery rate (FDR)-adjusted *P* < .05. AGA, appropriate-for-gestational age; BD, block design; BW-for-GA, birthweight-for-gestational age; FGD, fetal abdominal circumference growth deceleration; IQ, intelligence quotient; LGA, large-for-gestational age; MR, matrix reasoning; SGA, small-for-gestational age; Y, year; z-FAC, fetal abdominal circumference z-score.

Every 1 SD increase in fetal AC z-score from the 2nd to 3rd trimester was associated with higher non-verbal IQ (4.5 years) [β (95% CI), 1.79 (0.38, 3.20)] (*P *=* *.013, FDR-adjusted *P *=* *.099) (Fig. 1), with consistent direction and magnitude of associations for verbal IQ (4.5 years) [1.35 (-0.05, 2.74)] and secondary outcome measures—PPVT score (4 years) [1.61 (0.33, 2.89)], lollipop test score (4 years) [1.83 (0.87, 2.78)] (Supplementary Fig. S2).

Fetal AC growth deceleration was associated with lower non-verbal IQ (4.5 years) [–4.00 (–7.49, –0.51)] (*P *=* *.025, FDR-adjusted *P *=* *.099), with consistent associations for lollipop test score (4 years) [–2.85 (–5.07, –0.63)] and verbal IQ (4.5 years) [–3.12 (–6.40, 0.16)]. Fetal AC growth, whether as a continuous or categorical variable, were not associated with any cognition outcomes at age 7 years. Birthweight-for-gestational age z-scores and SGA (vs. AGA) were not consistently associated with any cognition outcomes.

### Postnatal growth

**Figure 2.**
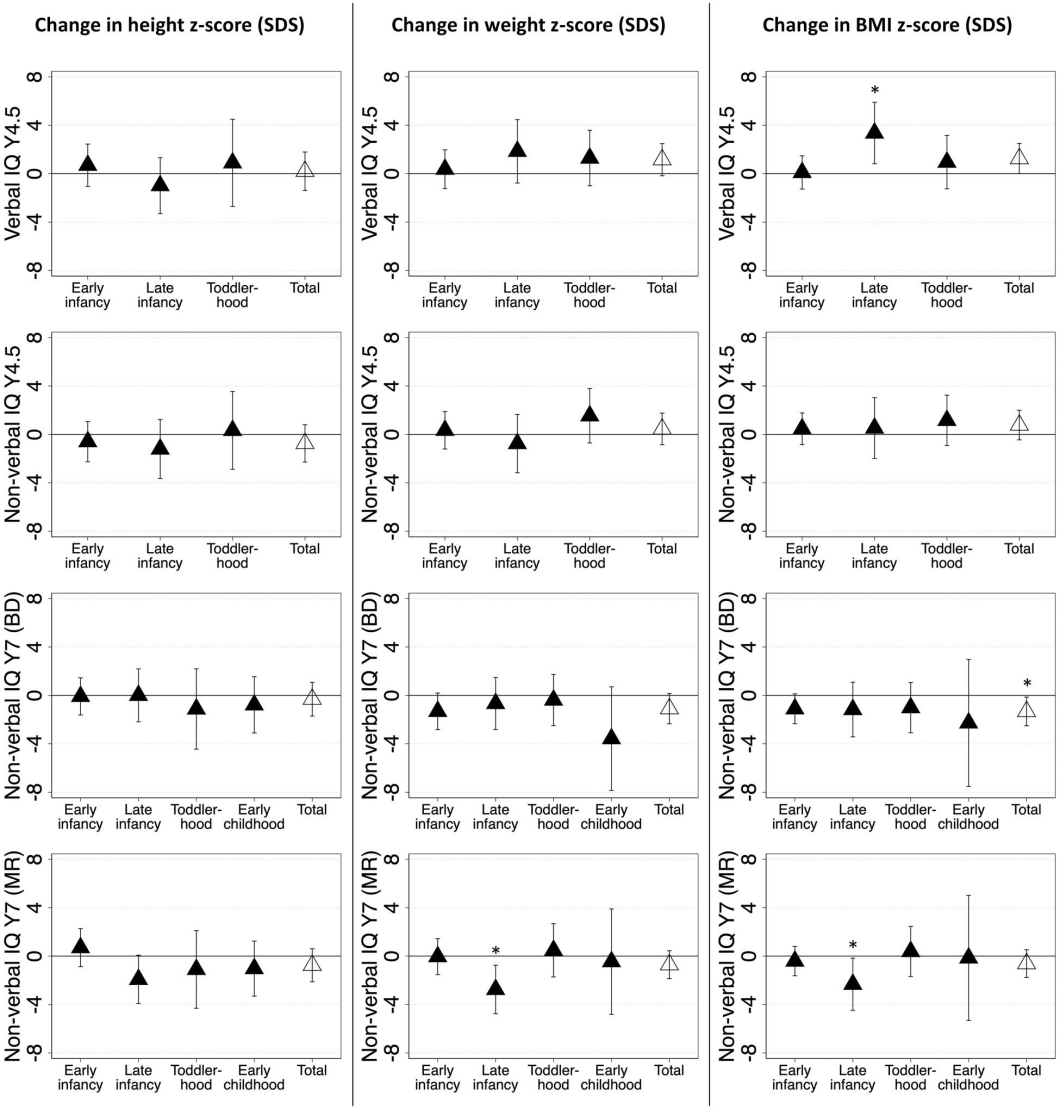
Associations between period-specific postnatal growth (height, weight, and body mass index) and cognition outcomes. The age periods include early infancy (0–4 months), late infancy (4–15 months), toddlerhood (15–37 months), and early childhood (37–84 months). Models are adjusted for parents’ education, parents’ height, household income, mother’s age, parity, ethnicity, pre-pregnancy body mass index, gestational tobacco exposure, depressive symptoms, child’s sex, gestational age, age at cognition measure, fetal abdominal growth, and postnatal growth at preceding age periods. In these coefficient plots, markers represent regression coefficients and error bars represent 95% confidence intervals, and asterisks represent *P* < .05. None of the associations had a false discovery rate (FDR)-adjusted *P* < .05. BD, block design; BMI, body mass index; IQ, intelligence quotient; MR, matrix reasoning; SDS, standard deviation score; Y, year. (As a change in height z-score by 1 SDS in early childhood is rare and leads to extreme beta coefficients, we reported the change in cognition scores per 0.1 SDS change in height z-score for the early childhood period.)

Every 1-SD unit increase in 0–7 years z-BMI growth was associated with lower non-verbal IQ (block design) (7 years) [–1.33 (–2.51, –0.14)] (*P *=* *.028, FDR-adjusted *P *=* *.208) (Fig. 2). Similar findings were seen when investigating z-weight in place of z-BMI. Investigating specific age periods, late infancy (4–15 months) z-BMI growth was associated with higher verbal IQ (4.5 years) [3.36 (0.82, 5.90)] (*P *=* *.010, FDR-adjusted *P *=* *.172) but lower non-verbal IQ (matrix reasoning) (7 years) [–2.32 (–4.48, –0.17)] (*P *=* *.035, FDR-adjusted *P *=* *.208). There were no consistent associations between z-height growth at any age period and cognition outcomes.

### Interaction between fetal and postnatal growth

**Figure 3.**
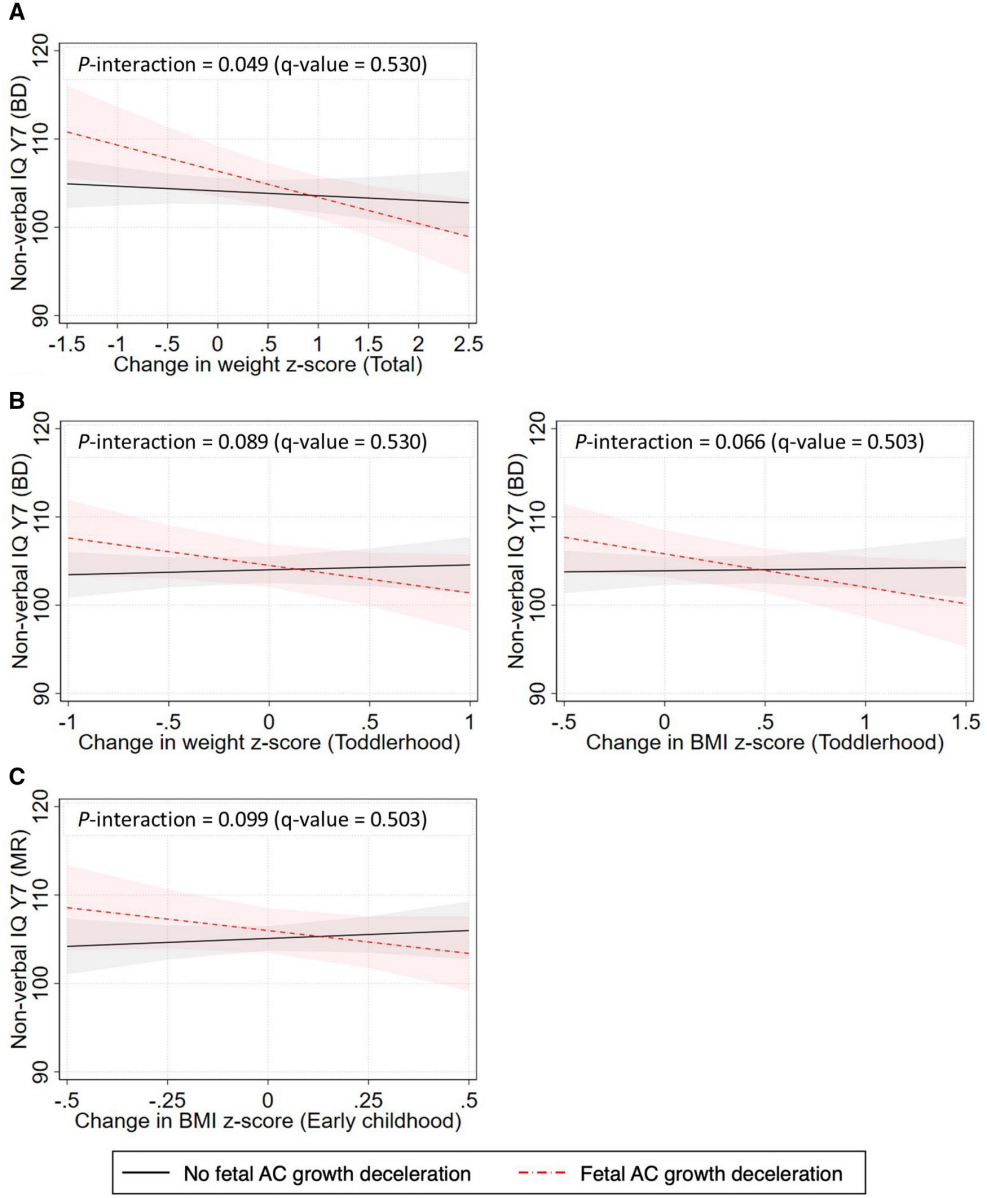
Interactions between fetal growth deceleration and postnatal growth on cognition outcomes from (A) 0–7 years, (B) 15–37 months, and (C) 37–84 months. Graphs show predicted values of cognition outcomes across the range of postnatal growth for children with and without fetal abdominal circumference growth deceleration, while holding all other covariates at the mean. Models are adjusted for parents’ education, parents’ height, household income, mother’s age, parity, ethnicity, pre-pregnancy body mass index, gestational tobacco exposure, depressive symptoms, child’s sex, gestational age, and age at cognition measure. Only models with *P*-interaction < .1 are shown. False discovery rate (FDR)-adjusted *P*-values (*q*-value) are presented in addition to unadjusted *P*-interaction values. AC, abdominal circumference; BD, block design; BMI, body mass index; IQ, intelligence quotient; MR, matrix reasoning; Y, year.

There was a potential interaction between fetal AC growth deceleration and 0–7 years z-weight growth in relation to non-verbal IQ (block design) (7 years) (*P*-interaction = .049, FDR-adjusted *P*-interaction = .530), which did not pass the FDR adjustment. Among children who experienced fetal AC growth deceleration, those with greater 0–7 years z-weight growth (2 SDS vs. 1 SDS increase in WHO weight z-score from 0 to 7 years old) had lower non-verbal IQ (block design) (7 years) [margins (95% CI), 100.4 (96.9, 103.9) vs. 103.4 (101.0, 105.8)] (Fig. 3). At z-weight gain of 2 SDS, those with (versus without) fetal AC growth deceleration had lower non-verbal IQ (block design) (7 years) [–2.6 (–7.1, 1.9)]. Consistent with this finding, among those with fetal AC growth deceleration, higher toddlerhood z-weight (*P*-interaction = .089, FDR-adjusted *P*-interaction = .530), and z-BMI (*P*-interaction = .066, FDR-adjusted *P*-interaction = .503) gain were associated with lower non-verbal IQ (block design) (7 years), while higher early childhood z-BMI gain (*P*-interaction = .099, FDR-adjusted *P*-interaction = .503) was associated with lower non-verbal IQ (matrix reasoning) (7 years).

**Figure 4.**
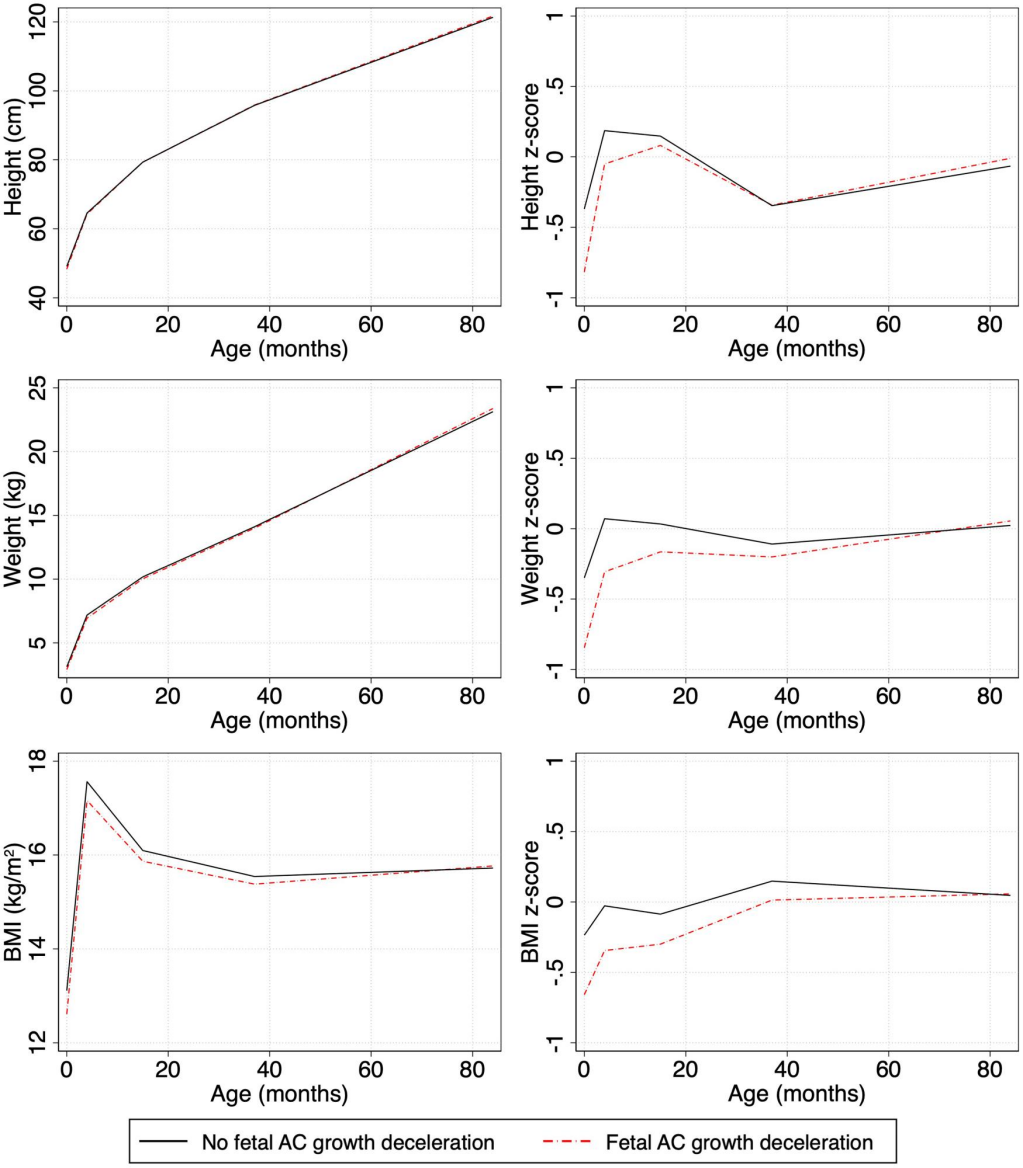
Postnatal growth trajectories and standardized World Health Organization z-score trajectories, visualized for children with fetal growth deceleration (dotted line) and without fetal growth deceleration (solid line). AC, abdominal circumference; BMI, body mass index.

Children with fetal AC growth deceleration caught up and even exceeded the average z-height, z-weight, and z-BMI of children without fetal AC growth deceleration by age 7 years (Fig. 4). Children with fetal AC growth deceleration had higher total height velocity (0.87 ± 0.06 vs. 0.86 ± 0.05 cm/month), especially in early infancy (3.99 ± 0.39 vs. 3.86 ± 0.38 cm/month). They also had higher total BMI velocity (0.04 ± 0.03 vs. 0.03 ± 0.03 kg/m^2^ per month), especially in late infancy (–0.12 ± 0.09 vs. –0.13 ± 0.09 kg/m^2^ per month) (Supplementary Table S4).

### Sensitivity analysis

We observed similar patterns of associations in complete case analysis (Supplementary Figs S3–S5), when using alternative cut-off points for defining fetal AC growth deceleration (Supplementary Fig. S6), and when additionally adjusting for father’s BMI (Supplementary Figs. S7–S9).

## Discussion

In our study, fetal AC growth deceleration from the second to third trimester occurred in 23.3% of term-born infants and was consistently associated with lower cognition scores at preschool age. No consistent associations were observed with birthweight-for-gestational age or SGA. This suggests the potential of longitudinal ultrasounds to identify and monitor children who had undergone fetal AC growth deceleration, even if they were not born preterm, low birthweight, or SGA. Contrary to our initial hypothesis, our study suggests no beneficial effect of increased postnatal weight, height, or BMI growth following fetal AC growth deceleration on child cognition. On average, children with fetal AC growth deceleration gradually ‘caught-up’ in weight, height, and BMI by age 7 years and seem to be on a trajectory set to surpass the weight, height, and BMI of children without fetal AC growth deceleration beyond age 7 years. Caution is warranted regarding 0–7 years weight gain, which was associated with lower non-verbal IQ at age 7 years, especially among children with fetal AC growth deceleration.

Consistent associations of fetal AC growth deceleration—but not SGA—with lower cognition scores at preschool age suggest that serial ultrasound measurements might be more sensitive than birthweight-for-gestational age in capturing poor fetal growth and neurodevelopmental alterations associated with it. A review of 14 studies similarly found no consistent associations between SGA/fetal growth restriction and neurocognitive outcomes among term-born children [30] - none of these studies investigated fetal AC growth. Differentiating fetal AC growth deceleration from SGA is clinically important as these two conditions might be linked to different perinatal/child outcomes [1, 2]. We focused on investigating fetal AC growth which was identified as the most promising individual biometry parameter for identifying fetal growth restriction [31], potentially with higher sensitivity compared to estimated fetal weight [32, 33]. Although a recent study found no associations between fetal AC z-score at gestational weeks 26–28 and neurodevelopment at age 2 years [34], fetal AC at a single timepoint is not equivalent to fetal AC growth deceleration. Worryingly, other studies have associated slower fetal AC growth with suboptimal perinatal/child outcomes—adverse neonatal outcomes and morbidity [7, 8], reduced feto-placental blood flow throughout pregnancy [6], poorer vision [6], and cardiometabolic risk [5, 9]. These suggest the emerging importance of monitoring fetal AC growth for early risk stratification.

Reassuringly, children with fetal AC growth deceleration had a gradual ‘catch-up’ in postnatal growth. By school age, there were no longer any associations between fetal AC growth deceleration and lower cognition scores. This is consistent with systematic reviews in term-born SGA children, which found positive associations between postnatal catch-up growth and cognition [12, 35]. Among 1957 term-born SGA infants from the Collaborative Perinatal Project, those with no catch-up weight gain had increased risk of having low IQ at age 7 years while those with appropriate catch-up weight gain—characterized by rapid catch-up to the 30th percentile by age 4 months and gradual growth to the 50th percentile by age 7 years—was not associated with low IQ [36]. This pattern of appropriate catch-up weight gain is remarkably similar to the postnatal weight trajectories observed among children with fetal AC growth deceleration in our study, which might explain the lack of association with lower cognitive scores by age 7 years. However, increased overall weight gain from 0 to 7 years was associated with lower non-verbal IQ especially among those with fetal AC growth deceleration—excessive weight z-score gain by 2 standard deviations from 0 to 7 years was associated with an approximately 4-point lower-than-average non-verbal IQ (block design). These associations seem to be driven by weight gain in late infancy and toddlerhood (4–37 months). Similarly, other studies found that greater weight-for-height at age 1–2 years [37], fat mass gain from age 4 months to 4 years [38], and late infancy (4–15 months) BMI gain [21] were associated with lower IQ scores. Therefore, it is important to ensure appropriate, but not excessive, catch-up weight gain among children with fetal AC growth deceleration.

The strengths of this study include the use of serial ultrasounds to measure fetal AC growth deceleration, detailed modelling of postnatal growth trajectories to study period-specific postnatal growth, investigation of cognitive outcomes at two timepoints, elucidation of interactions between fetal and postnatal growth, and careful adjustment for multiple confounders. We used internationally recognized INTERGROWTH-21st standards for fetal AC z-scores and clinically relevant cut-offs to define fetal AC growth deceleration, which can be applied to other studies for replication. We cautiously interpreted our findings by considering the consistency in direction of associations across various sensitivity analyses, biological plausibility, and consistency with literature. Limitations of the study include the use of two different tools (KBIT-2 and WASI-II) for cognitive assessment at ages 4.5 and 7 years, respectively. However, we ensured that both tests are well-validated [24, 39] and the matrix reasoning subtest of WASI-II is comparable to the KBIT-2 matrices subtest used to assess non-verbal IQ. Our study also lacked fetal Doppler data to further distinguish between pathological and non-pathological fetal growth restriction. As this distinction is beyond the scope of the current study, further studies are needed to explore both longitudinal fetal growth and Doppler measures to define pathological fetal growth restriction and investigate its impact on child outcomes. This study only included approximately 80% of participants from the original cohort; about 30% of participants had data on all three main outcomes (IQ scores at years 4.5 and 7), which might lead to selection bias as these participants differ in various baseline characteristics. However, we used inverse probability of censoring weighting in our main analyses to minimize this issue. Moreover, our complete case analyses showed similar results with our main analyses which further adds to the robustness of our study findings. There might be residual confounding due to the lack of adjustment for unmeasured confounders (e.g. quality of the home environment). Our study might not be generalizable to other populations (e.g. preterm infants, twins, populations with different rates of obesity). Additionally, a considerable proportion of excluded participants did not have father’s education data. This group of participants might have a different distribution in fathers’ education levels. The findings of our study need to be replicated in other cohorts.

In conclusion, it might be important for children with fetal AC growth deceleration to be followed up postnatally for early identification and prompt management of excessive weight gain, to help them achieve their full potential.

## Ethics approval

The National Healthcare Group Domain Specific Review Board (D/09/021) and SingHealth Centralized Institutional Review Board (2018/2767/D) approved this study and written informed consent was obtained from all study participants. This study conforms to the Declaration of Helsinki ethical standards.

## Supplementary Material

dyaf012_Supplementary_Data

## Data Availability

The data used in the manuscript are available on request, on approval by the GUSTO executive committee.

## Supplementary data

Conflict of interest: K.M.G. has received reimbursement for speaking at conferences sponsored by companies selling nutritional products and is part of an academic consortium that has received research funding from Abbott Nutrition, Nestec, BenevolentAI Bio Ltd, and Danone. S.Y.C. is part of an academic consortium that has received research support from Nestle SA and has received reimbursement for speaking at scientific meetings from Nestle Nutrition Institute. All other authors report no conflicts of interest.
