# Supplementary material for: Associations of fetal and postnatal growth trajectories with child cognition: the GUSTO cohort study
Source: Int J Epidemiol. 2025 Feb 13;54(1):dyaf012. doi: 10.1093/ije/dyaf012 (PMC11825177; doi:10.1093/ije/dyaf012)
Supplement: dyaf012_Supplementary_Data [file dyaf012_supplementary_data.zip › 58fdd_ije-2024-04-0491-File007.docx]

**SUPPLEMENTARY MATERIAL**

Table of Contents

[Supplementary Figure S1: Participant flowchart. 2](#_Toc188636945)

[Supplementary Figure S2: Associations of fetal and period-specific postnatal growth with additional cognition outcomes. 3](#_Toc188636946)

[Supplementary Figure S3: Associations between fetal growth and cognition outcomes (complete case analysis). 4](#_Toc188636947)

[Supplementary Figure S4: Associations between period-specific postnatal growth and cognition outcomes (complete case analysis). 5](#_Toc188636948)

[Supplementary Figure S5: Interactions between fetal growth deceleration and postnatal growth on cognition outcomes, visualized for children with fetal growth deceleration (red dotted line) and without fetal growth deceleration (black solid line) (complete case analysis). 6](#_Toc188636949)

[Supplementary Figure S6: Associations of fetal abdominal circumference growth with cognition outcomes, using alternative cut-off points for defining fetal abdominal circumference growth deceleration. 7](#_Toc188636950)

[Supplementary Figure S7: Associations between fetal growth and cognition outcomes, additionally adjusted for father’s body mass index. 8](#_Toc188636951)

[Supplementary Figure S8: Associations between period-specific postnatal growth (height, weight, body mass index) and cognition outcomes, additionally adjusted for father’s body mass index. 9](#_Toc188636952)

[Supplementary Figure S9: Interactions between fetal growth deceleration and postnatal growth on cognition outcomes, additionally adjusted for father’s body mass index. 10](#_Toc188636953)

[Supplementary Table S1: Model selection for postnatal growth trajectories. 11](#_Toc188636954)

[Supplementary Table S2: Characteristics of included and excluded participants. 12](#_Toc188636955)

[Supplementary Table S3: Characteristics of participants with all 3 main outcomes (IQ at Y4.5, Y7) measured compared to participants without. 13](#_Toc188636956)

[Supplementary Table S4: Growth of children with and without fetal abdominal circumference growth deceleration from linear spline mixed effect models. 15](#_Toc188636957)

Supplementary Figure S1: Participant flowchart.^[[1]](#footnote-1)^


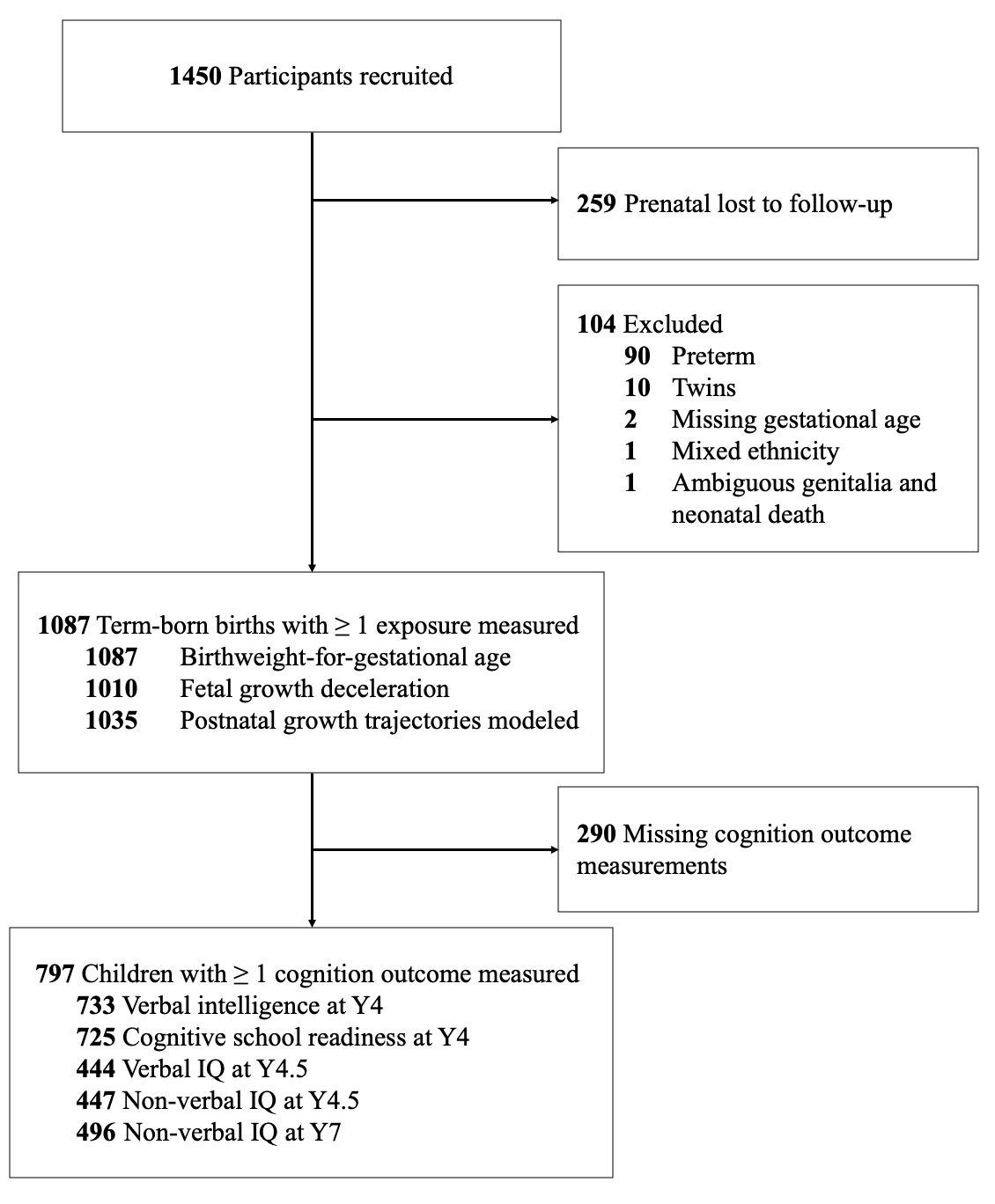


Supplementary Figure S2: Associations of fetal and period-specific postnatal growth with additional cognition outcomes. The age periods include early infancy (0-4 months), late infancy (4-15 months), toddlerhood (15-37 months), and early childhood (37-84 months). Models are adjusted for parents’ education, parents’ height, household income, mother’s age, parity, ethnicity, pre-pregnancy body mass index, gestational tobacco exposure, depressive symptoms, child’s sex, gestational age, age at cognition measure. Models for postnatal growth are additionally adjusted for fetal abdominal growth and postnatal growth at preceding age periods. In these coefficient plots, markers represent regression coefficients and error bars represent 95% confidence intervals, and asterisks represent *P*<0.05.^[[2]](#footnote-2)^


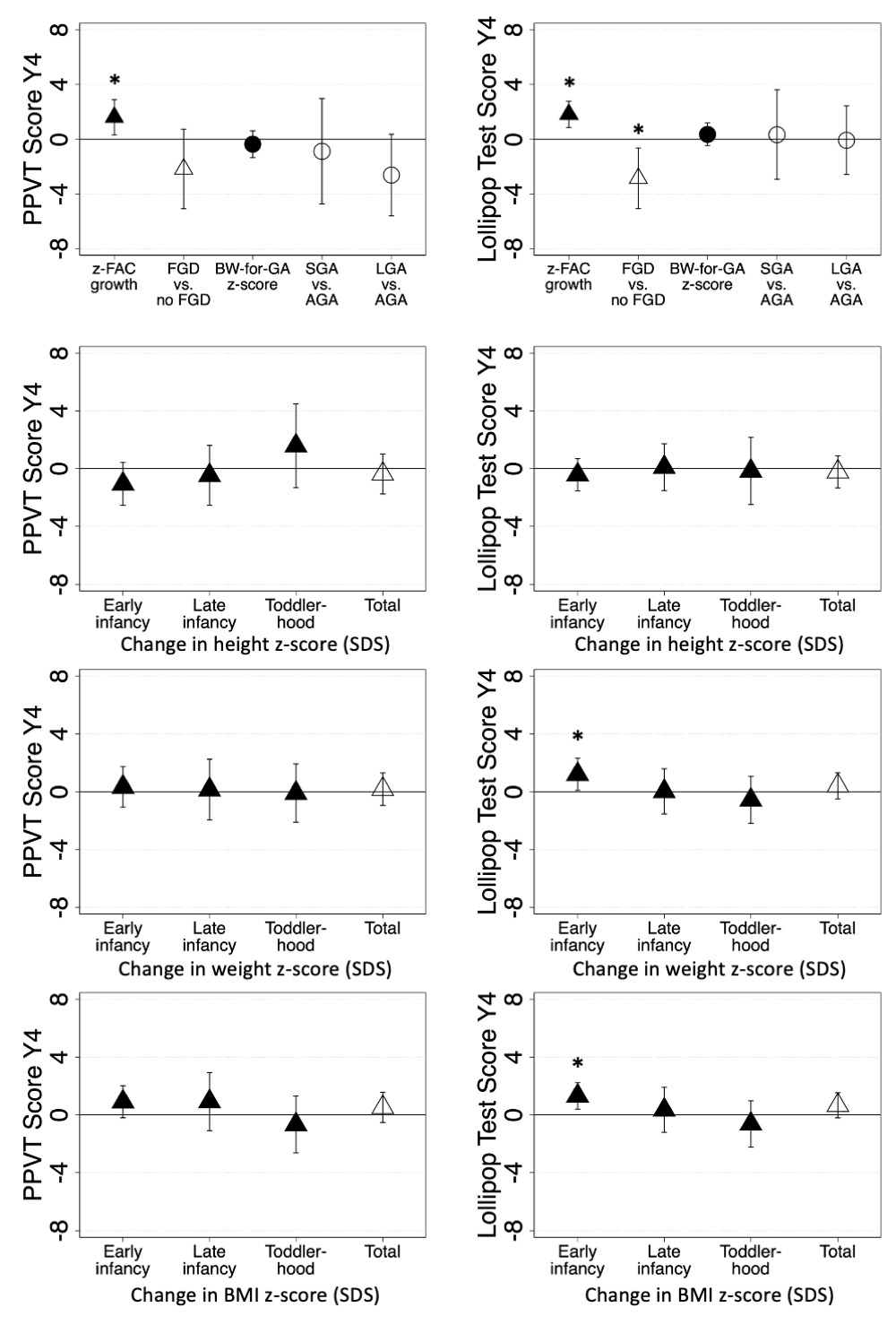


Supplementary Figure S3: Associations between fetal growth and cognition outcomes (complete case analysis). Models are adjusted for parents’ education, parents’ height, household income, mother’s age, parity, ethnicity, pre-pregnancy body mass index, gestational tobacco exposure, depressive symptoms, child’s sex, gestational age, and age at cognition measure. In these coefficient plots, markers represent regression coefficients and error bars represent 95% confidence intervals, and asterisks represent *P*<0.05.^[[3]](#footnote-3)^


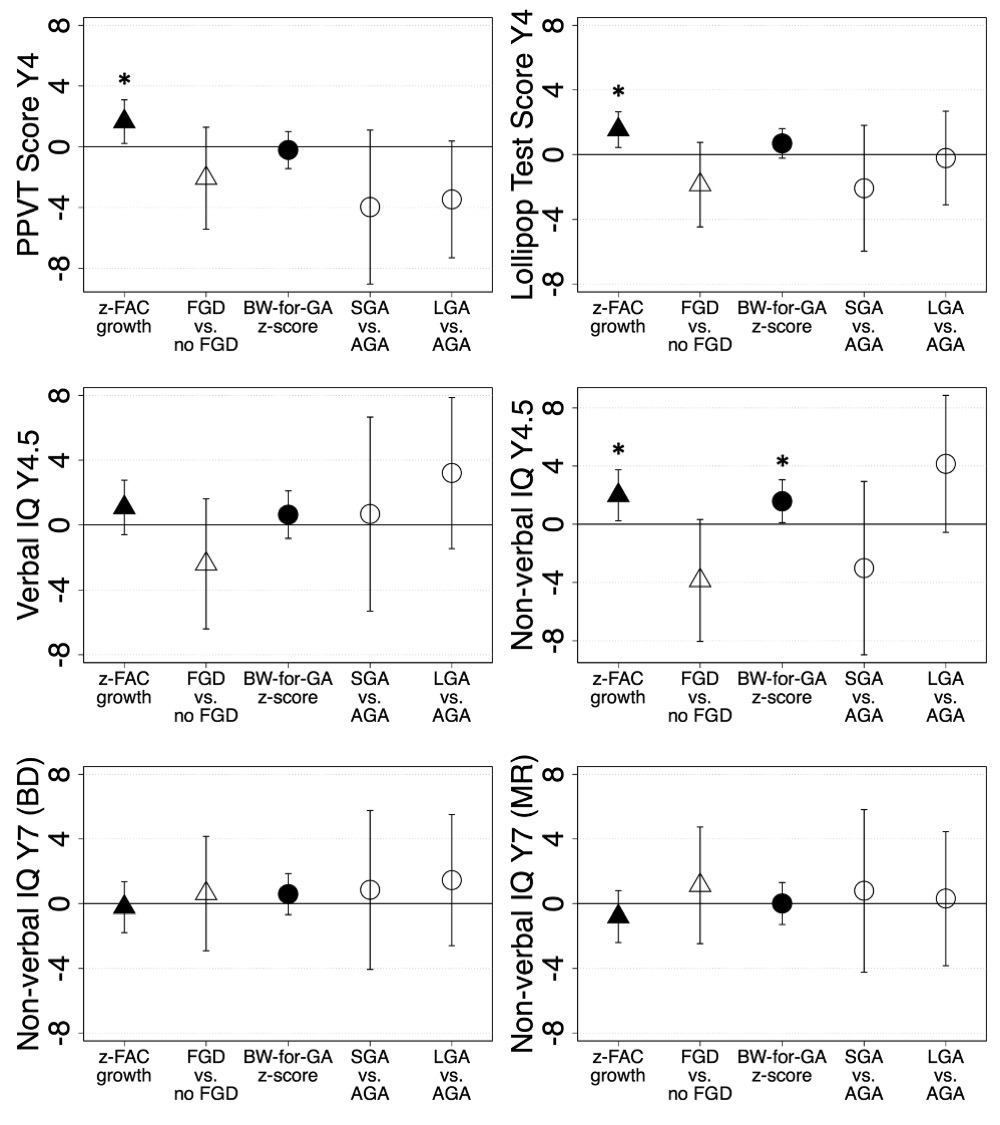


Supplementary Figure S4: Associations between period-specific postnatal growth and cognition outcomes (complete case analysis). The age periods include early infancy (0-4 months), late infancy (4-15 months), toddlerhood (15-37 months), and early childhood (37-84 months). Models are adjusted for parents’ education, parents’ height, household income, mother’s age, parity, ethnicity, pre-pregnancy body mass index, gestational tobacco exposure, depressive symptoms, child’s sex, gestational age, age at cognition measure, fetal abdominal growth, and postnatal growth at preceding age periods. In these coefficient plots, markers represent regression coefficients and error bars represent 95% confidence intervals, and asterisks represent *P*<0.05.^^[[4]](#footnote-4)^^^[[5]](#footnote-5)^


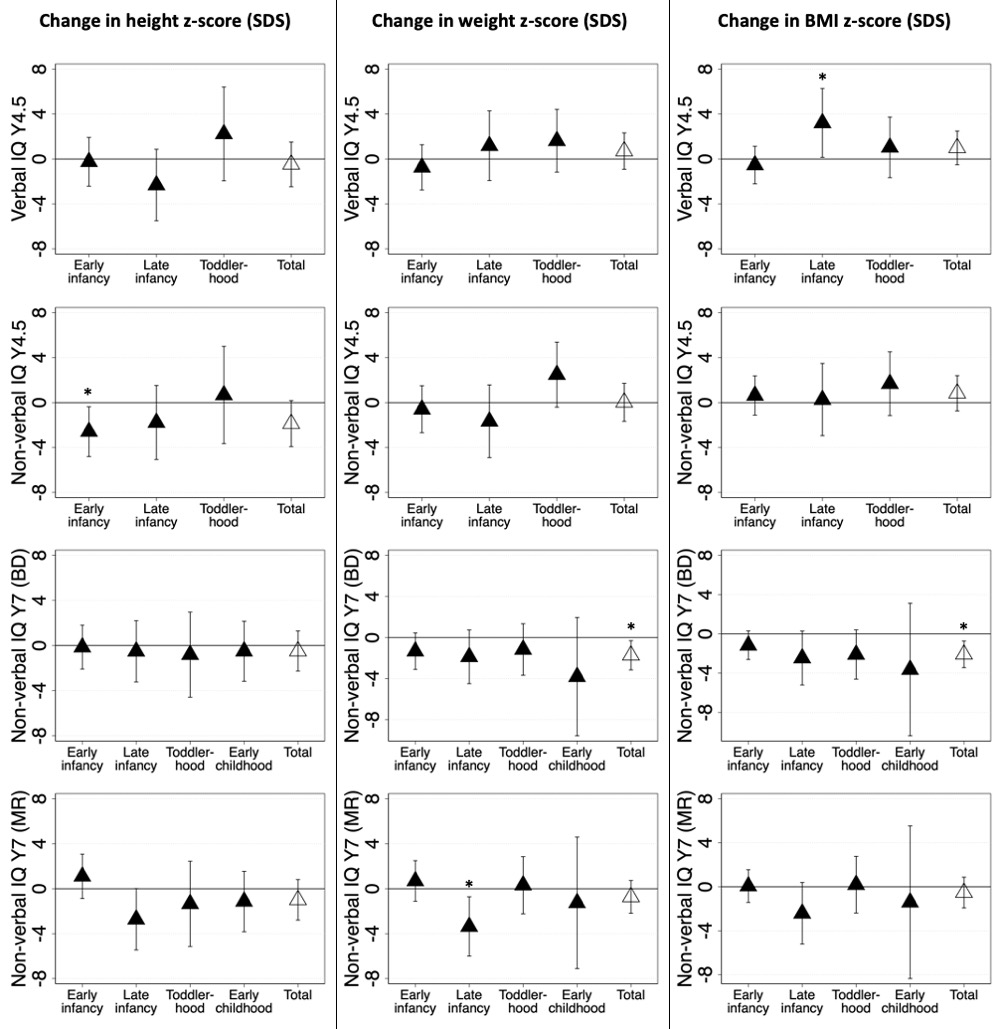


Supplementary Figure S5: Interactions between fetal growth deceleration and postnatal growth on cognition outcomes, visualized for children with fetal growth deceleration (red dotted line) and without fetal growth deceleration (black solid line) (complete case analysis). Graphs show predicted values of cognition outcomes across the range of postnatal growth for children with and without fetal abdominal circumference growth deceleration, while holding all other covariates at the mean. Models are adjusted for parents’ education, parents’ height, household income, mother’s age, parity, ethnicity, pre-pregnancy body mass index, gestational tobacco exposure, depressive symptoms, child’s sex, gestational age, and age at cognition measure. Only models with significant interaction are shown.^[[6]](#footnote-6)^


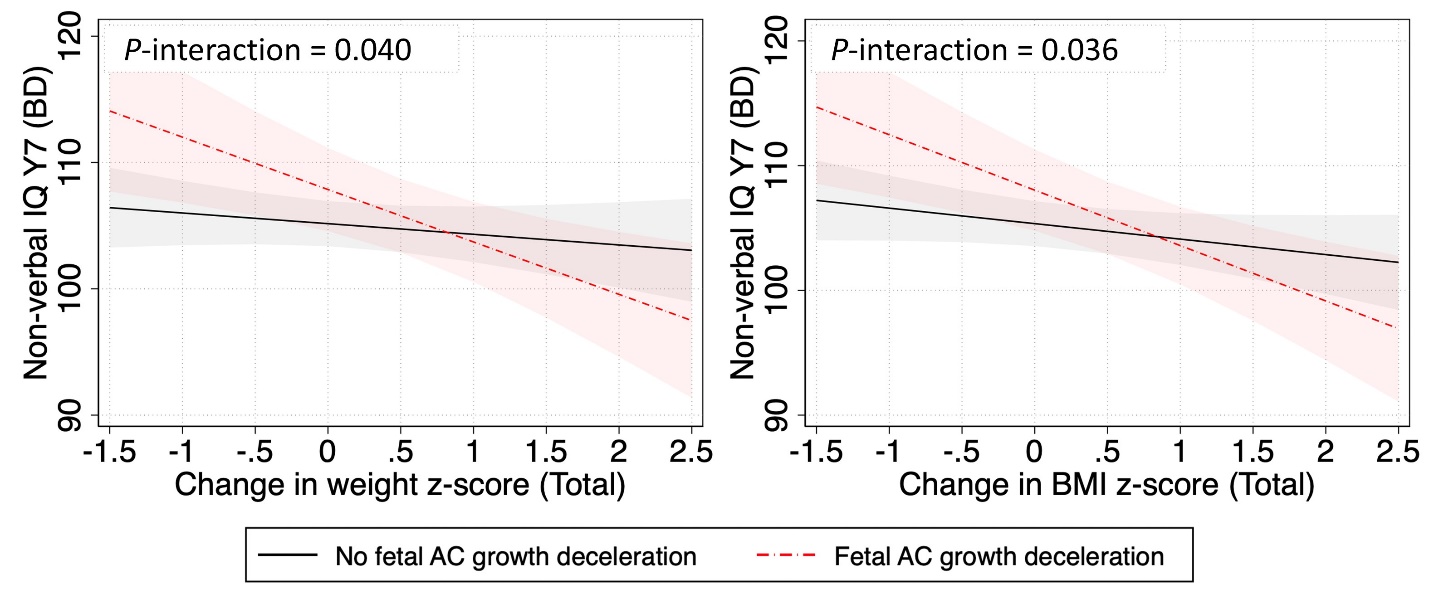


Supplementary Figure S6: Associations of fetal abdominal circumference growth with cognition outcomes, using alternative cut-off points for defining fetal abdominal circumference growth deceleration. Models are adjusted for parents’ education, parents’ height, household income, mother’s age, parity, ethnicity, pre-pregnancy body mass index, gestational tobacco exposure, depressive symptoms, child’s sex, gestational age, and age at cognition measure. In these coefficient plots, markers represent regression coefficients and error bars represent 95% confidence intervals, and asterisks represent *P*<0.05.^[[7]](#footnote-7)^


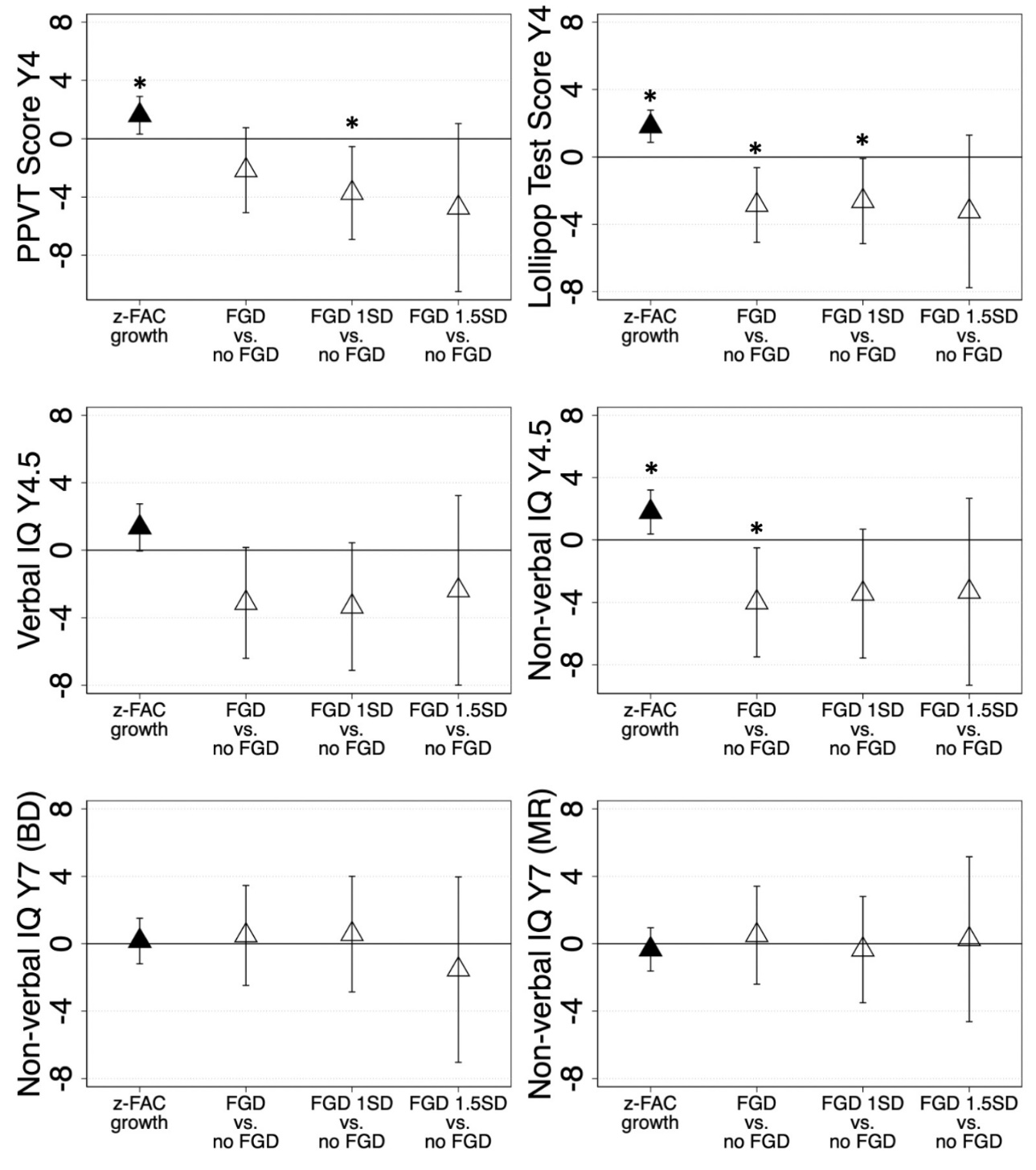


Supplementary Figure S7: Associations between fetal growth and cognition outcomes, additionally adjusted for father’s body mass index. Models are adjusted for parents’ education, parents’ height, household income, mother’s age, parity, ethnicity, pre-pregnancy body mass index, father’s body mass index, gestational tobacco exposure, depressive symptoms, child’s sex, gestational age, and age at cognition measure. In these coefficient plots, markers represent regression coefficients and error bars represent 95% confidence intervals, and asterisks represent *P*<0.05.^[[8]](#footnote-8)^


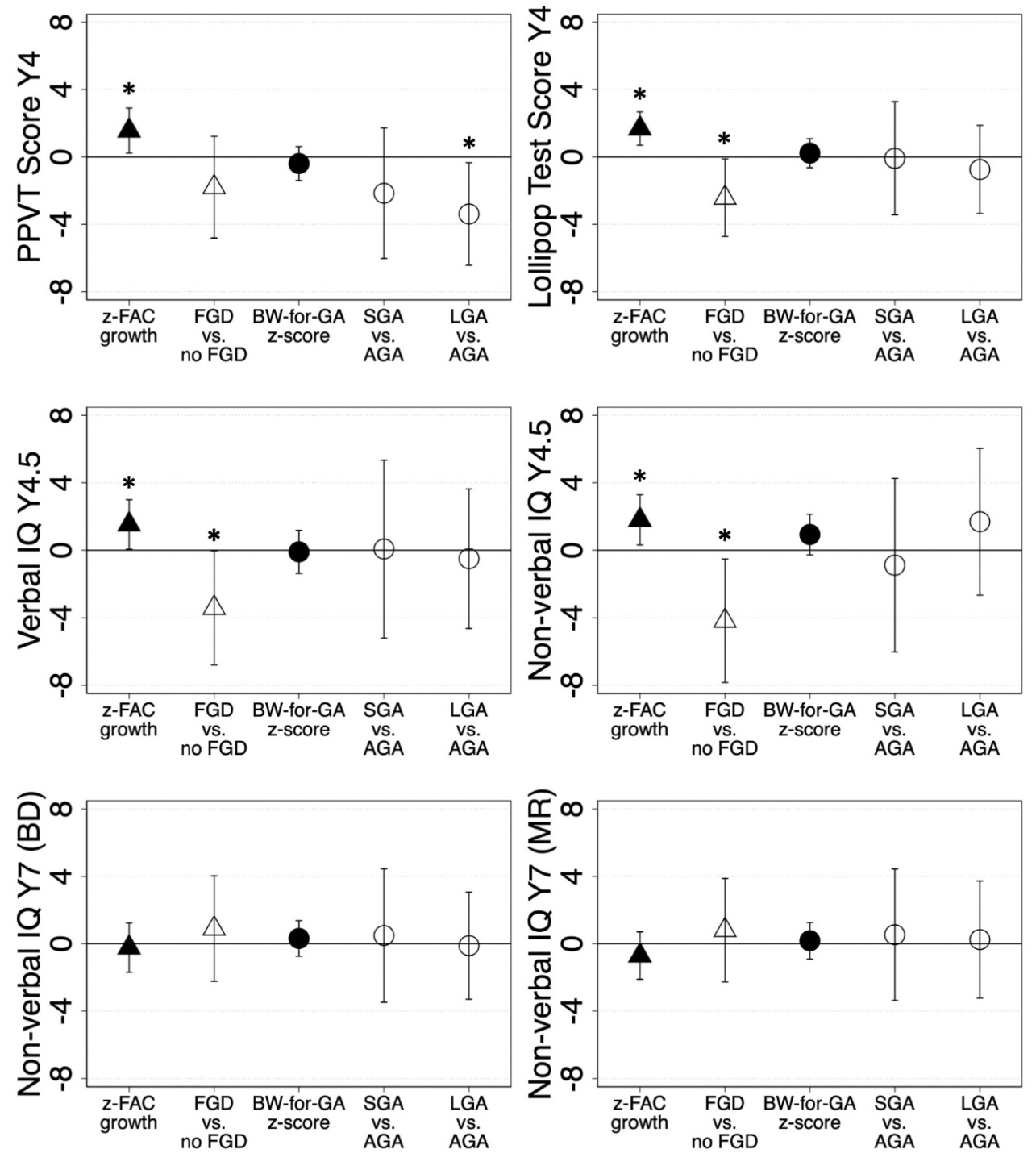


Supplementary Figure S8: Associations between period-specific postnatal growth (height, weight, body mass index) and cognition outcomes, additionally adjusted for father’s body mass index. The age periods include early infancy (0-4 months), late infancy (4-15 months), toddlerhood (15-37 months), and early childhood (37-84 months). Models are adjusted for parents’ education, parents’ height, household income, mother’s age, parity, ethnicity, pre-pregnancy body mass index, father’s body mass index, gestational tobacco exposure, depressive symptoms, child’s sex, gestational age, age at cognition measure, fetal abdominal growth, and postnatal growth at preceding age periods. In these coefficient plots, markers represent regression coefficients and error bars represent 95% confidence intervals, and asterisks represent *P*<0.05.^^[[9]](#footnote-9)^^^[[10]](#footnote-10)^


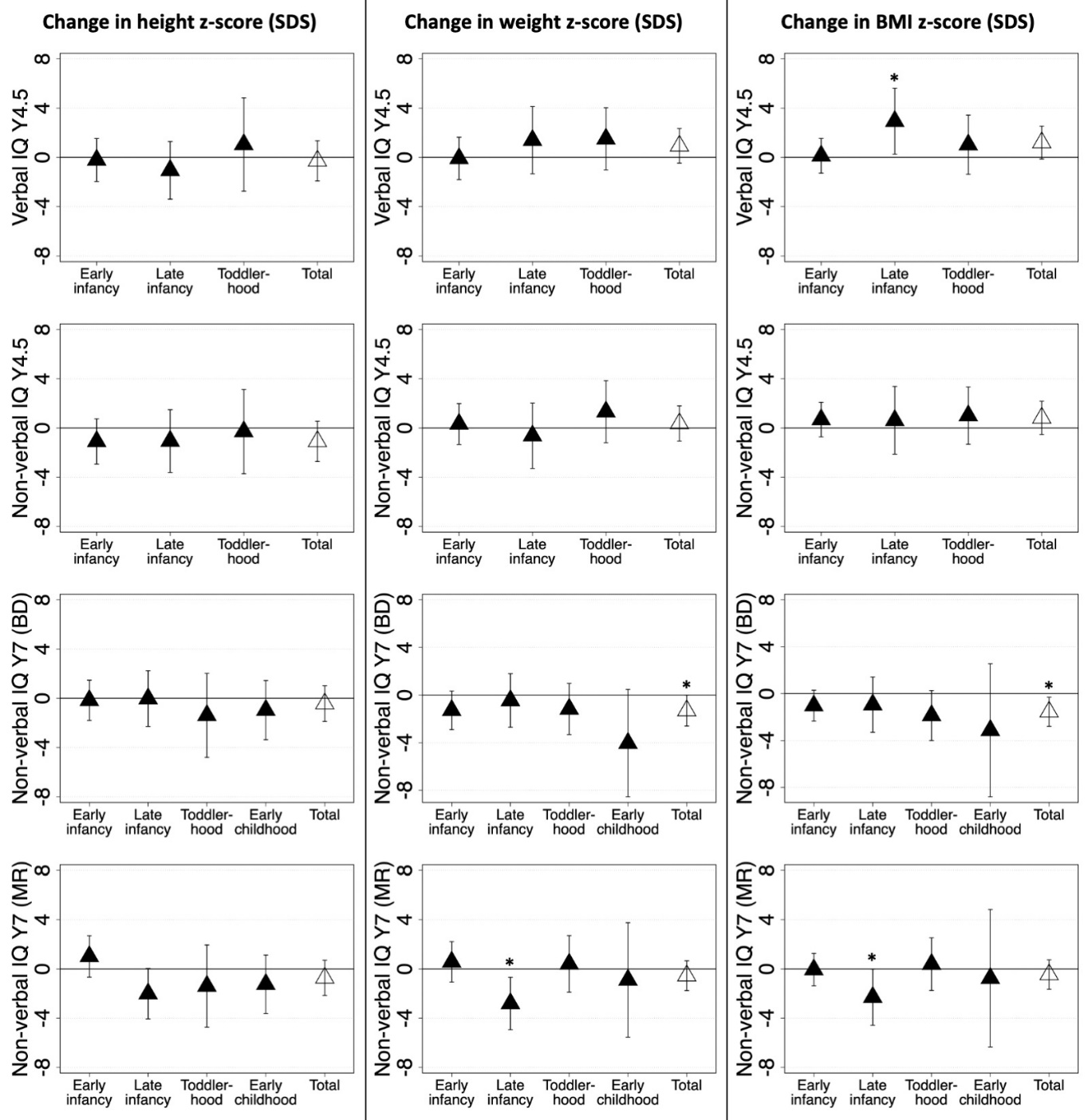


Supplementary Figure S9: Interactions between fetal growth deceleration and postnatal growth on cognition outcomes, additionally adjusted for father’s body mass index. The graph shows predicted values of cognition outcomes across the range of postnatal growth for children with and without fetal abdominal circumference growth deceleration, while holding all other covariates at the mean. Models are adjusted for parents’ education, parents’ height, household income, mother’s age, parity, ethnicity, pre-pregnancy body mass index, father’s body mass index, gestational tobacco exposure, depressive symptoms, child’s sex, gestational age, and age at cognition measure. Only models with significant interaction are shown.^[[11]](#footnote-11)^


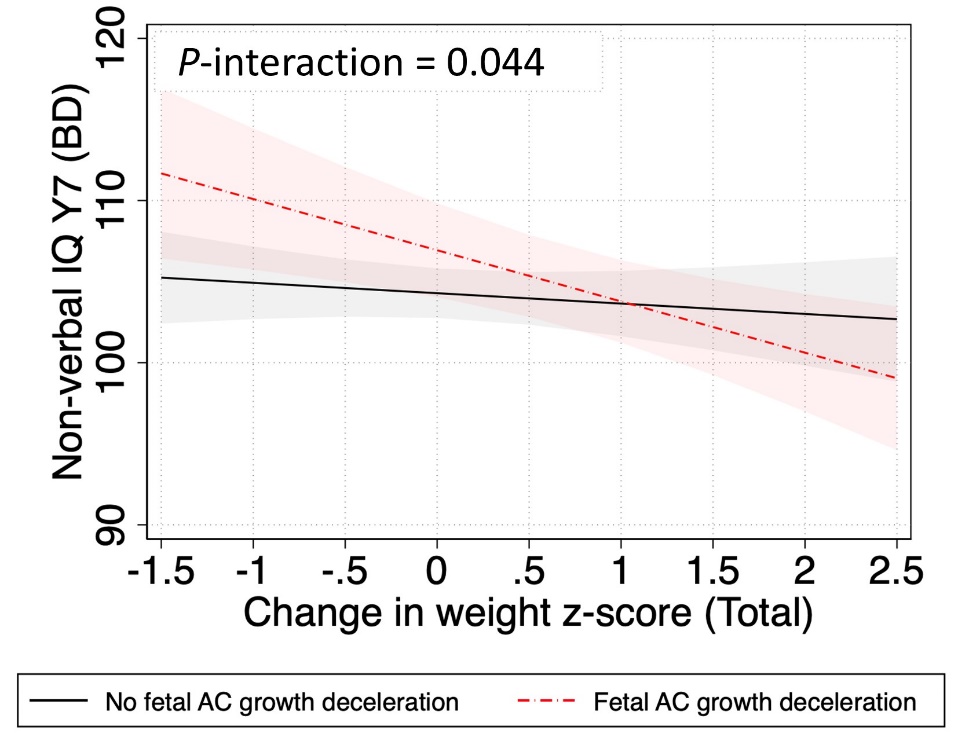


Supplementary Table S1: Model selection for postnatal growth trajectories.^[[12]](#footnote-12)^

| Growth parameter | Approaches | Knot positions (months) | Bayesian information criterion (BIC) | Model selected |
| --- | --- | --- | --- | --- |
| Height | Specified *a priori* based on literature | 3, 12, 36 | 65961.51 |  |
|  | Specified *a priori* based on literature: Project Viva study | 4, 15, 37 | 65063.35 | Selected – lowest BIC |
|  | Equidistant percentiles of age distribution (25^th^, 50^th^, 75^th^ percentile) | 3, 16, 55 | 66031.84 |  |
|  | Equidistant percentiles of age distribution (33^rd^, 66^th^ percentile) | 9, 42 | 73719.65 |  |
| Weight | Specified *a priori* based on literature | 3, 12 | 42667.5 |  |
|  | Specified *a priori* based on literature: Project Viva study | 4, 15, 37 | 38783.91 | Selected – for consistency with height and BMI trajectories. Second lowest BIC |
|  | Equidistant percentiles of age distribution (25^th^, 50^th^, 75^th^ percentile) | 3, 16, 55 | 37420.12 |  |
|  | Equidistant percentiles of age distribution (33^rd^, 66^th^ percentile) | 9, 42 | 42191.19 |  |
| Body mass index | Specified *a priori* based on literature: Project Viva study | 4, 15, 37 | 47325.45 | Selected – lowest BIC |
|  | Equidistant percentiles of age distribution (25^th^, 50^th^, 75^th^ percentile) | 3, 16, 55 | 47648.8 |  |
|  | Equidistant percentiles of age distribution (33^rd^, 66^th^ percentile) | 9, 42 | 55964.7 |  |

Supplementary Table S2: Characteristics of included and excluded participants.^[[13]](#footnote-13)^

| Characteristics, mean ± SD / *n* (%) | Included (N=797) | Excluded (N=290) | *P*-value |
| --- | --- | --- | --- |
| Mother's age, years (n=1087) | 30.9 ± 5.0 | 30.1 ± 5.2 | 0.029 |
| Ethnicity |  |  |  |
| Chinese | 456 (57.2%) | 156 (53.8%) | 0.545 |
| Malay | 201 (25.2%) | 76 (26.2%) |  |
| Indian | 140 (17.6%) | 58 (20.0%) |  |
| Mother's education |  |  |  |
| Not college graduate | 504 (63.2%) | 193 (66.6%) | 0.183 |
| College graduate | 286 (35.9%) | 90 (31.0%) |  |
| Data missing | 7 (0.9%) | 7 (2.4%) |  |
| Father's education |  |  |  |
| Not college graduate | 380 (47.7%) | 58 (20.0%) | 0.655 |
| College graduate | 261 (32.7%) | 36 (12.4%) |  |
| Data missing | 156 (19.6%) | 196 (67.6%) |  |
| Monthly household income |  |  |  |
| Low (S$0-3999) | 334 (41.9%) | 120 (41.4%) | 0.138 |
| Mid (S$4000-6000) | 177 (22.2%) | 76 (26.2%) |  |
| High (>S$6000) | 236 (29.6%) | 69 (23.8%) |  |
| Data missing | 50 (6.3%) | 25 (8.6%) |  |
| Parity |  |  |  |
| Primiparous | 362 (45.4%) | 133 (45.9%) | 0.897 |
| Multiparous | 435 (54.6%) | 157 (54.1%) |  |
| Mother's height, cm (n= 1064) | 158.2 ± 5.7 | 158.7 ± 5.6 | 0.189 |
| Father's height, cm (n=851) | 170.8 ± 6.2 | 170.8 ± 6.2 | 0.997 |
| Pre-pregnancy BMI, kg/m^2^ (n=991) | 22.8 ± 4.4 | 22.7 ± 4.6 | 0.718 |
| Maternal tobacco exposure |  |  |  |
| No exposure | 476 (59.7%) | 166 (57.2%) | 0.677 |
| Secondhand exposure | 258 (32.4%) | 101 (34.8%) |  |
| Current smoker | 20 (2.5%) | 6 (2.1%) |  |
| Data missing | 43 (5.4%) | 17 (5.9%) |  |
| Depressive symptoms during pregnancy |  |  |  |
| EPDS score < 13 | 679 (85.2%) | 245 (84.5%) | 0.978 |
| EPDS score ≥ 13 | 92 (11.5%) | 33 (11.4%) |  |
| Data missing | 26 (3.3%) | 12 (4.1%) |  |
| Sex |  |  |  |
| Male | 387 (48.6%) | 127 (43.8%) | 0.164 |
| Female | 410 (51.4%) | 163 (56.2%) |  |
| Gestational age, weeks (n=1087) | 39.0 ± 1.0 | 39.0 ± 1.0 | 0.328 |
| BW-for-GA z-score (n=1087) | 0.20 ± 1.18 | 0.05 ± 1.21 | 0.072 |
| Appropriate-for-gestational age | 585 (73.4%) | 227 (78.3%) | 0.232 |
| Small-for-gestational age | 83 (10.4%) | 27 (9.3%) |  |
| Large-for-gestational age | 129 (16.2%) | 36 (12.4%) |  |
| Fetal AC z-score change from 2^nd^ to 3^rd^ trimester (n=1010) | 0.01 ± 0.99 | -0.01 ± 0.92 | 0.798 |
| No fetal AC growth deceleration | 557 (69.9%) | 204 (70.3%) | 0.640 |
| Fetal AC growth deceleration | 186 (23.3%) | 63 (21.7%) |  |
| Data missing | 54 (6.8%) | 23 (7.9%) |  |

Supplementary Table S3: Characteristics of participants with all 3 main outcomes (**IQ scores at years 4.5 and 7**) measured compared to participants without.^[[14]](#footnote-14)^

| Characteristics, mean ± SD / *n* (%) | Participants with all 3 main outcomes measured (N=339) | Participants without all 3 main outcomes measured (N=748) | *P*-value |
| --- | --- | --- | --- |
| Mother's age, years (n=1087) | 30.9 ± 5.2 | 30.5 ± 5.0 | 0.265 |
| Ethnicity |  |  |  |
| Chinese | 193 (56.9%) | 419 (56.0%) | 0.056 |
| Malay | 97 (28.6%) | 180 (24.1%) |  |
| Indian | 49 (14.5%) | 149 (19.9%) |  |
| Mother's education |  |  |  |
| Not college graduate | 231 (68.1%) | 466 (62.3%) | 0.064 |
| College graduate | 104 (30.7%) | 272 (36.4%) |  |
| Data missing | 4 (1.2%) | 10 (1.3%) |  |
| Father's education |  |  |  |
| Not college graduate | 184 (54.3%) | 254 (34.0%) | <0.001 |
| College graduate | 87 (25.7%) | 210 (28.1%) |  |
| Data missing | 68 (20.1%) | 284 (38.0%) |  |
| Monthly household income |  |  |  |
| Low (S$0-3999) | 156 (46.0%) | 298 (39.8%) | 0.211 |
| Mid (S$4000-6000) | 75 (22.1%) | 178 (23.8%) |  |
| High (>S$6000) | 88 (26.0%) | 217 (29.0%) |  |
| Data missing | 20 (5.9%) | 55 (7.4%) |  |
| Parity |  |  |  |
| Primiparous | 156 (46.0%) | 339 (45.3%) | 0.831 |
| Multiparous | 183 (54.0%) | 409 (54.7%) |  |
| Mother's height, cm (n= 1064) | 158.1 ± 5.7 | 158.4 ± 5.6 | 0.456 |
| Father's height, cm (n=851) | 170.3 ± 6.4 | 171.0 ± 6.1 | 0.112 |
| Pre-pregnancy BMI, kg/m^2^ (n=991) | 23.0 ± 4.6 | 22.7 ± 4.4 | 0.291 |
| Maternal tobacco exposure |  |  |  |
| No exposure | 178 (52.5%) | 464 (62.0%) | 0.012 |
| Secondhand exposure | 126 (37.2%) | 233 (31.1%) |  |
| Current smoker | 12 (3.5%) | 14 (1.9%) |  |
| Data missing | 23 (6.8%) | 37 (4.9%) |  |
| Depressive symptoms during pregnancy |  |  |  |
| EPDS score < 13 | 278 (82.0%) | 646 (86.4%) | 0.015 |
| EPDS score ≥ 13 | 51 (15.0%) | 74 (9.9%) |  |
| Data missing | 10 (2.9%) | 28 (3.7%) |  |
| Sex |  |  |  |
| Male | 162 (47.8%) | 352 (47.1%) | 0.824 |
| Female | 177 (52.2%) | 396 (52.9%) |  |
| Gestational age, weeks (n=1087) | 39.0 ± 1.0 | 39.0 ± 1.0 | 0.901 |
| BW-for-GA z-score (n=1087) | 0.18 ± 1.23 | 0.15 ± 1.18 | 0.689 |
| Appropriate-for-gestational age | 237 (69.9%) | 575 (76.9%) | 0.035 |
| Small-for-gestational age | 44 (13.0%) | 66 (8.8%) |  |
| Large-for-gestational age | 58 (17.1%) | 107 (14.3%) |  |
| Fetal AC z-score change from 2^nd^ to 3^rd^ trimester (n=1010) | 0.00 ± 0.97 | 0.00 ± 0.97 | 0.969 |
| No fetal AC growth deceleration | 237 (69.9%) | 524 (70.1%) | 0.319 |
| Fetal AC growth deceleration | 86 (25.4%) | 163 (21.8%) |  |
| Data missing | 16 (4.7%) | 61 (8.2%) |  |

Supplementary Table S4: Growth of children with and without fetal abdominal circumference growth deceleration from linear spline mixed effect models.^[[15]](#footnote-15)^

| Characteristics, mean ± SD | Fetal AC growth deceleration | No fetal AC growth deceleration | *P*-value |
| --- | --- | --- | --- |
| Change in height, cm per month |  |  |  |
| Early infancy (0-4m) | 3.99 ± 0.39 | 3.86 ± 0.38 | <0.001 |
| Late infancy (4-15m) | 1.37 ± 0.13 | 1.34 ± 0.13 | 0.008 |
| Toddlerhood (15-37m) | 0.75 ± 0.08 | 0.75 ± 0.07 | 0.410 |
| Early childhood (37-84m) | 0.55 ± 0.04 | 0.54 ± 0.04 | 0.030 |
| Total (0-84m) | 0.87 ± 0.06 | 0.86 ± 0.05 | <0.001 |
| Change in weight, kg per month |  |  |  |
| Early infancy (0-4m) | 1.01 ± 0.13 | 1.01 ± 0.14 | 0.526 |
| Late infancy (4-15m) | 0.28 ± 0.05 | 0.27 ± 0.05 | 0.015 |
| Toddlerhood (15-37m) | 0.18 ± 0.06 | 0.18 ± 0.06 | 0.659 |
| Early childhood (37-84m) | 0.20 ± 0.09 | 0.19 ± 0.08 | 0.181 |
| Total (0-84m) | 0.24 ± 0.06 | 0.24 ± 0.06 | 0.182 |
| Change in BMI, kg/m^2^ per month |  |  |  |
| Early infancy (0-4m) | 1.14 ± 0.38 | 1.11 ± 0.38 | 0.375 |
| Late infancy (4-15m) | -0.12 ± 0.09 | -0.13 ± 0.09 | 0.016 |
| Toddlerhood (15-37m) | -0.02 ± 0.04 | -0.03 ± 0.04 | 0.265 |
| Early childhood (37-84m) | 0.01 ± 0.04 | 0.00 ± 0.03 | 0.062 |
| Total (0-84m) | 0.04 ± 0.03 | 0.03 ± 0.03 | 0.002 |

1. IQ, intelligence quotient; Y, year. [↑](#footnote-ref-1)
2. AGA, appropriate-for-gestational age; BMI, body mass index; BW-for-GA, birthweight-for-gestational age; FGD, fetal abdominal circumference growth deceleration; LGA, large-for-gestational age; PPVT, Peabody Picture Vocabulary Test; SDS, standard deviation score; SGA, small-for-gestational age; Y, year; z-FAC, fetal abdominal circumference z-score. [↑](#footnote-ref-2)
3. AGA, appropriate-for-gestational age; BD, block design; BW-for-GA, birthweight-for-gestational age; FGD, fetal abdominal circumference growth deceleration; IQ, intelligence quotient; LGA, large-for-gestational age; MR, matrix reasoning; PPVT, Peabody Picture Vocabulary Test; SGA, small-for-gestational age; Y, year; z-FAC, fetal abdominal circumference z-score. [↑](#footnote-ref-3)
4. As a change in height z-score by 1 SDS in early childhood is rare and leads to extreme beta coefficients, we reported the change in cognition scores per 0.1 SDS change in height z-score for the early childhood period. [↑](#footnote-ref-4)
5. BD, block design; BMI, body mass index; IQ, intelligence quotient; MR, matrix reasoning; SDS, standard deviation score; Y, year. [↑](#footnote-ref-5)
6. AC, abdominal circumference; BD, block design; BMI, body mass index; IQ, intelligence quotient; Y, year. [↑](#footnote-ref-6)
7. BD, block design; FGD, fetal abdominal circumference growth deceleration; FGD 1SD, decrease in z-FAC by at least 1 SD; FGD 1.5SD, decrease in z-FAC by at least 1.5 SD; IQ, intelligence quotient; MR, matrix reasoning; PPVT, Peabody Picture Vocabulary Test; SD, standard deviation; Y, year; z-FAC, fetal abdominal circumference z-score. [↑](#footnote-ref-7)
8. AGA, appropriate-for-gestational age; BD, block design; BW-for-GA, birthweight-for-gestational age; FGD, fetal abdominal circumference growth deceleration; IQ, intelligence quotient; LGA, large-for-gestational age; MR, matrix reasoning; PPVT, Peabody Picture Vocabulary Test; SGA, small-for-gestational age; Y, year; z-FAC, fetal abdominal circumference z-score. [↑](#footnote-ref-8)
9. As a change in height z-score by 1 SDS in early childhood is rare and leads to extreme beta coefficients, we reported the change in cognition scores per 0.1 SDS change in height z-score for the early childhood period. [↑](#footnote-ref-9)
10. BD, block design; BMI, body mass index; IQ, intelligence quotient; MR, matrix reasoning; SDS, standard deviation score; Y, year. [↑](#footnote-ref-10)
11. AC, abdominal circumference; BD, block design; IQ, intelligence quotient; Y, year. [↑](#footnote-ref-11)
12. BIC, Bayesian information criterion. [↑](#footnote-ref-12)
13. AC, abdominal circumference; BMI, body mass index; BW-for-GA, birthweight-for-gestational age; EPDS, Edinburgh Postnatal Depression Scale; SD, standard deviation. [↑](#footnote-ref-13)
14. AC, abdominal circumference; BMI, body mass index; BW-for-GA, birthweight-for-gestational age; EPDS, Edinburgh Postnatal Depression Scale; SD, standard deviation. [↑](#footnote-ref-14)
15. AC, abdominal circumference; BMI, body mass index; SD, standard deviation. [↑](#footnote-ref-15)
